# “Same same” but different? Exploring the impact of perceived organizational support at the school and teacher levels on teachers’ job engagement and organizational citizenship behavior

**DOI:** 10.3389/fpsyg.2022.1067054

**Published:** 2022-12-29

**Authors:** Chuan-Chung Hsieh, Wei-Cheng Chien, Hung-Chin Yen, Hui-Chieh Li

**Affiliations:** ^1^Department of Education and Learning Technology, National Tsing Hua University, Hsinchu City, Taiwan; ^2^Research Center for Educational System and Policy, National Academy for Educational Research, New Taipei City, Taiwan; ^3^Department of Graphics Arts and Communtions, National Taiwan Normal University, Taipei City, Taiwan; ^4^Department of Nursing, School of Nursing, National Taipei University of Nursing and Health Sciences, Taipei City, Taiwan; ^5^Chaoliao Elementary School, Kaohsiung City, Taiwan; ^6^Center for Teacher Education, National Tsing Hua University, Hsinchu City, Taiwan

**Keywords:** job engagement, organizational citizenship behavior, school organizational support, structural equation modeling, multilevel structural equation modeling

## Abstract

All countries in the world are currently trying to implement educational reform, which increases the additional workload of teachers. It is more important to discuss how to inspire teachers’ enthusiasm for educational reform from the perspective of organizational support (OS). Previous research on OS was limited to perceived organizational support (POS), but in recent years group-level OS has been considered the most promising. There is no study comparing POS and group-level OS in education, and therefore this study explored the relationships between OS, job engagement (JE) and organizational citizenship behavior (OCB) in an educational context. In particular, OS was examined at the individual-level (POS) and the aggregated group-level (school organizational support, SOS). Analysis was performed using structural equation modeling (SEM) at both single and multiple levels. SEM results showed direct and positive relationships of individual-level POS with both JE and OCB. Moreover, JE is directly and positively related to OCB and plays the partial mediating role of the indirect and positive impact of POS on OCB. Multilevel structural equation modeling (MSEM) analysis revealed direct and positive relationships of SOS with teachers’ JE, which was directly and positively related to their OCB. While SOS had no significant positive relationship with OCB, it did have a positive impact on OCB through the mediation of JE. Comparison between SEM and MSEM results revealed the change in effect of OS on OCB from significant to insignificant, thus implying full mediation effect of JE when SOS is considered.

## Introduction

Countries around the world have been introducing and implementing educational reforms at different levels, with Taiwan being no exception. Curriculum amendments and increased use of educational technology not only pose challenges but also add extra workload to front-line teachers. In face of these professional challenges and work pressure, teachers of today have to be more resourceful and work even harder. Needless to say, educational reforms bring changes in the school organization. Hence, relevant organizational theories can be used as a reference for schools to comprehend, absorb, and adapt to these changes. In particular, the organizational support theory (OST) provides a mechanism for understanding the mutual commitment between organization members and the organization. Drawing on Blau’s social exchange theory and Gouldner’s norm of reciprocity, OST holds that members when given positive resources and attention would have their socio-emotional needs satisfied and hence feel obligated to help the organization reach its goals (Caesens and Stinglhamber, 2020). In Taiwan, the education system promises and provides sufficient job security to elementary school teachers; yet, motivations and incentives seem to be lacking. Organizational atmosphere of the school plays a critical role in motivating teachers to take up more duties and make extra efforts. On the basis of OST, this study aims to explore how school organizations can inspire teachers to have a stronger zeal for education and motivate them to contribute to educational reforms with concerted efforts.

Prior empirical research on OST has mainly analyzed and discussed its central psychological construct, perceived organizational support (POS; Baran et al., 2012). POS refers to the degree to which employees believe their work organization values their contributions and cares about their well-being (Eisenberger et al., 1986). Syahril et al. (2022) stated that POS is defined as members’ perception of the degree to which the organization provides support to the employees and the degree to which the organization is prepared to give assistance when needed. Findings from meta-analysis studies have revealed that POS is positively related to positive orientation toward the organization and affective organizational commitment, job performance and organizational citizenship behavior, subjective well-being in terms of job satisfaction and self-efficacy, organization-based self-esteem, and work-family balance (Aselage and Eisenberger, 2003; Kurtessis et al., 2017). With POS, employees feel obligated to reciprocate the organization’s support, more committed to the organization, and a greater desire to help the organization to succeed. In the post-COVID-19 era, how to improve employee performance to assist organizational development is an important topic. POS has a greater impact on employees’ work performance under COVID-19 (To and Huang, 2022), which shows its important research value. The study of Bernarto et al. (2020) also pointed out that teachers’ POS has a positive impact on job satisfaction and life satisfaction. Furthermore, POS appears to have stronger positive outcomes in Eastern cultures than in Western cultures (Eisenberger et al., 2020). Therefore, for Taiwanese teachers, POS is an indispensable and important assistance in the educational environment under the post-COVID-19 era.

In recent years, advances in multilevel research methods have led to breakthroughs in analytical approaches. Through data analytics, individual-level variables that used to reflect individual employee’s behavior or perception of the organization can now be aggregated as group/organization-level variables. Hence, concepts previously of individual levels, such as POS, can now be examined variables at the group or organization level, such as collective commitment (Raineri, 2017; Bal and Boehm, 2019) and group-level organizational citizenship behavior (Choi and Sy, 2010; Bakar and McCann, 2016). Such approach not only expands traditional research but is also becoming a new trend in organizational research. Especially in the POS, the studies in recent years have begun to examine the influence of group/organizational-level perceptions of organizational support, which has surpassed almost all individual-level POS; group-level organizational support (OS) is more regarded as the most promising field of recent research (Eisenberger et al., 2020). For example, Wang et al. (2011) found that increases in benevolence and decreases in authority in CEO communications were associated with managers’ positive attitudes at group-level OS. Berson et al. (2008) also found that group-level OS was positively correlated with CEO benevolent values and was also positively correlated with employee satisfaction. Wallace et al. (2006) found that group-level OS led to an enhanced organizational safety climate that would contribute to the reduction of accidents. González-Romá et al. (2009) found that group-level OS was positively correlated with team performance. These findings suggest that instructing leaders to convey positive appraisals of the contributions of their employees as a whole and the groups within them may broadly improve employees’ positive attitudes, performance, and well-being. Take the case of POS of teachers in schools for example. On one hand, it reflects individual teacher’s perception of the principal or the school as a whole; on the other hand, teachers of the same school work under the same principal or organization and theoretically should have similar perception. That is to say, there should be greater homogeneity in POS of teachers serving the same school. Therefore, aggregating the POS at the individual level of teachers from the same school into group-level OS can broaden the original POS research framework. Specifically, this approach is the major focus of the present study.

As mentioned above, POS is closely related to many positive organizational behaviors or mentalities. On the basis of positive psychology, job engagement (JE) is an emerging research topic that has attracted much attention. Also known as job engagement and employee engagement, JE refers to an individual’s willingness to invest positive cognitive, emotional, and physical energies into their work roles and to dedicate persistence and resilience in pursuing task performance (Christian et al., 2011). Of note is that while JE and similar concepts such as organizational commitment and job involvement are all related to identification and engagement in organizational work roles, JE puts greater emphasis on occupational health psychology or work-related well-being of employees (Bakker et al., 2008). Rich et al. (2010) reported the positive relationships of JE with task performance and organization citizenship behavior (OCB), highlighting the positive impact of job engagement on job performance. Contemporary education environment not only requires teachers to perform well in their teaching role, but also attaches great importance to their educational zeal and professionalism. Hence, JE as an antecedent of job performance and its related impacts are also topics that merit examination.

Moreover, organization citizenship behavior (OCB) is also an important aspect of employee performance worthy of investigation. Organ (1988) defines OCB as “individual behavior that is discretionary, not directly or explicitly recognized by the formal reward system, and that in the aggregate promotes the effective functioning of the organization.” According to Kim and Park (2022), the behaviors demanded by organizations today are not only in-role behaviors, doing the work as stated in the job description, but also extra-role behaviors, that is, contributing additional roles to accomplish the work of the organization. The OST theory offers a clear explanation for the psychological mechanism behind employees’ OCB. Specifically, POS reveals the employees’ view of how much their organization values their contribution and cares about their well-being (Eisenberger et al., 1986). In accord with the principles of social exchange and the norm of reciprocity, employees perceiving organizational support in the form of concern, appreciation, and affirmation tend to give in return more positive feedback and better performance, displaying OCBs (Rhoades and Eisenberger, 2002). Kurtessis et al. (2017) meta-analysis found that POS was positively correlated with in-role performance, OCB directed toward the organization and toward individuals. Such an outcome could be expected given OST’s assertion that POS would lead to a perceived obligation to help the organization achieve its goals and also would raise the expectation that performance will be rewarded. The meta-analysis also found that POS was more strongly correlated with OCB directed toward the organization than with OCB toward individuals (Eisenberger et al., 2020). Emerging education model expects teachers to break through the stereotypes of textbook teaching, using diverse means and manners to promote students’ learning progress and achieve educational goals. Inevitably, teachers would have to make extra effort and take on roles beyond the job description. Hence, exploring how to enhance OCB among teachers through group-level school organizational support (SOS) would be of significance to current organizational research in education.

In addition to OCBs, reciprocation of POS can also be seen in employees’ affective organizational commitment and job performance (Eisenberger et al., 2001). Research on organizational behavior has discovered that job engagement, job involvement, and organizational commitment are empirically distinct constructs, reflecting different aspects of work attachment (Hallberg and Schaufeli, 2006). Meta-analytic results showed that moderate positive correlation with POS and job involvement (Rhoades and Eisenberger, 2002). In view of this finding, the present research postulates that POS would also have a positive impact on JE.

In this study, POS of individual elementary school teachers in Taiwan are aggregated into a group-level variable, namely SOS, in the multilevel research framework to examine its impact on JE and OCB. Constrained by laws and regulations, public elementary schools in Taiwan cannot directly provide monetary incentives, such as bonus or salary rise, to teachers to motivate better job performance. Hence, exploring the contribution of organizational support from school to enhancement in teachers’ performance should be of relevance and significance. As mentioned above, the school is an educational organization and POS of teachers serving in the same school should be of higher consistency. Providing organizational support to teachers can enhance overall job engagement and increase OCBs among the staff, thus contributing to better educational achievement. With such presumption, this study aims to investigate in depth the relationships between SOS, JE, and OCB.

## Literature

### Perceived organizational support

Perceived organizational support (POS) is an important concept that explains the social exchange relationship between the organization and employees and the principle of reciprocity (Settoon et al., 1996). This concept helps explain the relationships as well as cognitive and emotional processes within the organization (Dulac et al., 2008). According to the definition of Eisenberger et al. (1986), POS is indicative of an individual member’s perception of the organization as a whole, how much the organization values the member’s contribution and how important the member is to the organization.

To date, individual-level POS has been found to have positive association with desirable workplace outcomes including high performance, high commitment, and low deviance (Harris and Kacmar, 2018) and interactive effects on affective commitment to the organization (Casimir et al., 2014; Gaudet and Tremblay, 2017). Moreover, while POS enhances job satisfaction (Cullen et al., 2014), it reduces turnover intention (Wayne et al., 1997; Ekrot et al., 2018). Related studies in the field of education obtained similar findings. Affective commitment and job satisfaction of physical educators are found to be positively related to their POS (Richards et al., 2020), while relationship and task conflicts at workplace are negatively related to POS of Belgian teachers (Caesens et al., 2019).

On the other hand, common views or shared perceptions of individual members of an organization can be aggregated into a general appraisal of the work climate, reflecting the overall impression about an organization (James and James, 1989). This perspective justifies the aggregation of individual-level POS into group-level OS, and such approach has been adopted by some recent studies. For instance, Li et al. (2017) aggregated individual-level POS to reflect the organizational support climate. Their study with cross-level design reported the double-edged moderating effect, highlighting that organizational support may not always have uniformly positive effects on affiliative OCB. Moreover, the multilevel analysis conducted by Chang et al. (2020) also found positive relationship between group-level OS and work performance of physical education teachers.

It is worth noting that the above-mentioned studies all measured group-level OS using the scale developed by Eisenberger et al. (1986). Hence, the nature of the aggregated group-level variable should be relatively uniform and consistent. Nevertheless, compared with stable results obtained using individual-level POS, the effects of group-level OS remain to be further clarified.

### Job engagement

Job engagement (JE) is defined as an individual’s willingness to invest positive cognitive, emotional, and physical energies into their work roles and is an important predictor of work performance (Christian et al., 2011). Consequences of high JE include happiness and job satisfaction rather than pressure from excessive work involvement or commitment (Rich et al., 2010). Hence, the concept of JE is an important topic in current research on positive organizational behavior.

Not only does JE inspire new insights in workplace psychology and behavior, its positive impact on work-related attitude and organizational conduct has also been proved in related research. Shantz et al. (2016) found that higher JE will reduce turnover intentions and deviant behaviors directed toward the organization, and such negative relationship is moderated by POS. Karanika-Murray et al. (2015) also found evidence on the mediating role of JE on job satisfaction enhanced by organizational identification. As mentioned above, Chang et al. (2014) reported positive relationship between POS and work performance. Li et al. (2012) evidenced that enhancement in job performance was mediated by job engagement.

Similar findings were obtained in studies conducted in the field of education. Job engagement was found to play a mediating role in the positive association between a supportive school climate and enhancement in knowledge creation practices of career and technical education teachers in the United States (Song et al., 2013). In the Netherlands, job engagement was also found to be positively related to job performance of starting teachers (Bakker and Bal, 2010) and OCB of secondary school teachers (Runhaar et al., 2013). Taken together, current findings evidenced the positive relationship of JE with work behaviors and attitudes in work environment of different fields.

### Organizational citizenship behavior

Organizational citizenship behavior (OCB), as defined by Organ (1988), refers to the discretionary behavior of an individual, neither defined by a job description nor recognized by the formal reward system, but in aggregate would be beneficial to organizational operations and performance. Such organizational construct is alternatively called extra-role behavior or contextual performance. While OCB can be manifested in many forms, there are several dimensions commonly adopted in related studies (Bragger et al., 2005; Somech and Ron, 2007). The five common dimensions are (1) altruism, voluntary assistance offered to fellow members on organizationally relevant tasks; (2) conscientiousness, acts that go beyond the minimal role requirements of the organization; (3) sportsmanship, willingness to tolerate inevitable inconveniences and less-than-ideal situations without complaining; (4) courtesy, preventing work-related problems with others; and (5) civic virtue, responsive, constructive involvement in the life of the organization.

Reforms and innovations in education have changed and expanded the traditional roles and responsibilities of teachers. Besides teaching, teachers are expected to take on additional duties and perform further tasks beneficial to both students and the school. Somech and Ron (2007) pointed out that the success of schools fundamentally depends on teachers’ willingness to go above and beyond the call of duty. That is, the responsibility of teachers is not only limited to the fixed and regular teaching duties, but should also involve assisting and promoting school development. The call for teachers to perform beneficial acts reveals the importance of OCB to both management and improvement of school.

Related studies on OCB of teachers reveal not only its close association with their job satisfaction and work efficacy (Jimmieson et al., 2010), but also its positive impact on enhancing students’ performance and quality of life as well as improving school image (Oplatka, 2009). As highlighted by Bogler and Somech (2004), OCB provides the organization with additional resources and eliminates the need for expensive formal mechanisms otherwise crucial to successful restructuring processes. In view of OCB as the key to organizational success, research on OCB of teachers has received considerable attention in the field of education.

### Theoretical model

**Figure 1:**
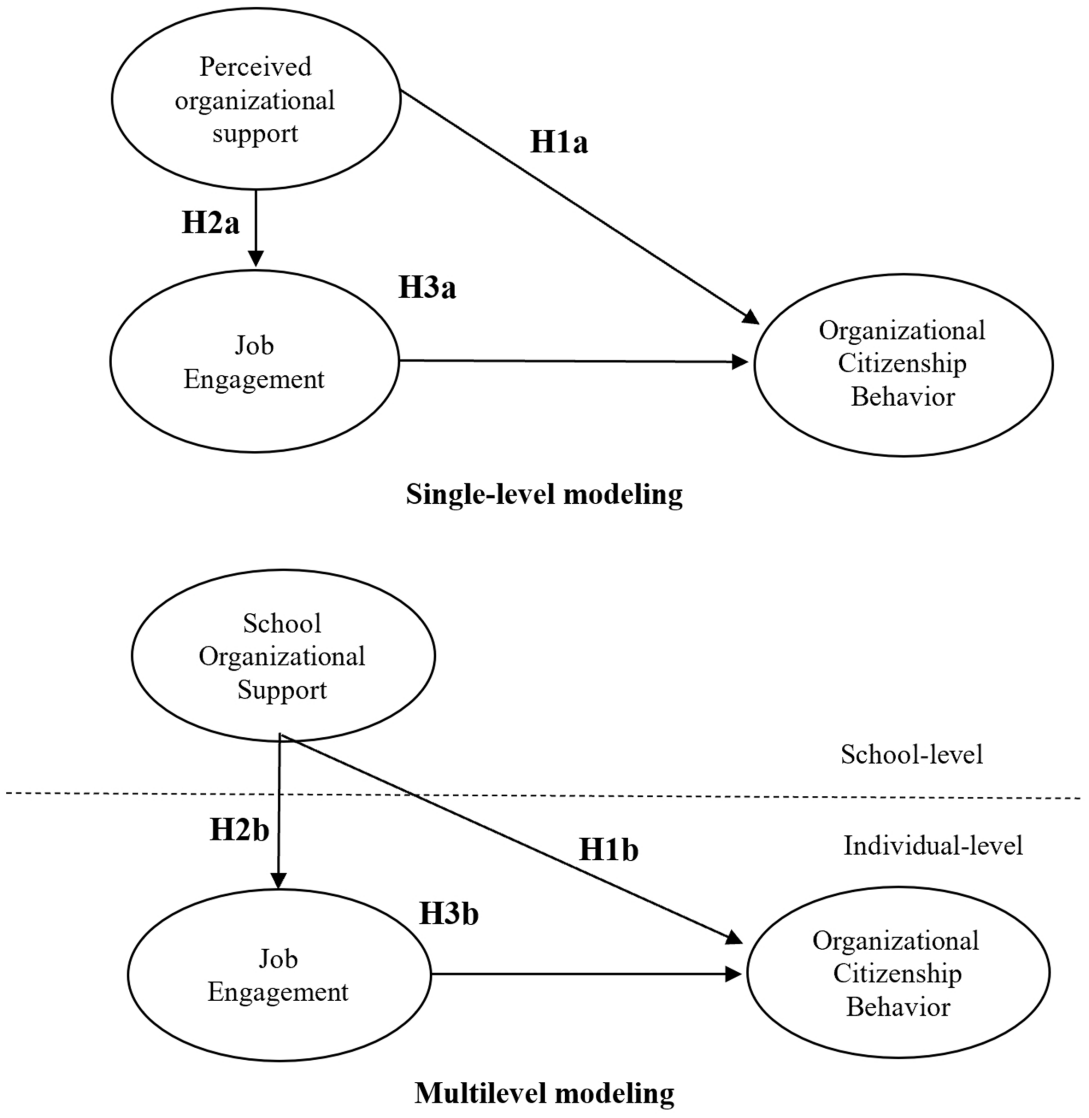
Theoretical model.

This study proposes a multilevel theoretical model for the relationships between group-level SOS, individual-level JE, and individual-level OCB. As illustrated in Figure 1, POS is not only an antecedent of both JE and OCB, but also has a positive impact on OCB through the mediation of JE. Of note is that POS, as the predictive variable, is analyzed at both individual teachers’ level and the aggregated school’s level. Not only can comparison between the two levels be made, results obtained can also bring new insight as related research using such approach is relatively scarce.

### SOS and individual-level OCB

This study used OST as the theoretical basis for examining POS. According to social exchange principles and norm of reciprocity, members perceiving support from the organization they belong will exhibit OCB in return. As the employees, when they are supported by the organization, they have an obligation to give back with their positive behavior. According to OST theory, if the employees perceive higher value, care, and support from the organization (such as POS), then they will get more rewards by exhibiting positive behaviors, thus developing a higher level of OCB (Spector and Che, 2014; Azim and Dora, 2016). Wahyuningrat and Rusmawan (2022) studied the impact of POS on OCB with job satisfaction as a mediating variable. The results of the study showed that POS has a positive impact on OCB. In their repeated assessment on POS and extra-role performance at a three-year interval, Chen et al. (2009) found evidence that POS leads to extra-role performance. Their results are echoed by those obtained by Muhammad (2014), Islam et al. (2014), Li et al. (2017), Zhang et al. (2017), and Ahmad and Zafar (2018) on the significant and positive relationship between POS and OCB. Wayne et al. (1997) in their study found that POS was strongly positively correlated with citizenship behavior at both the individual and organizational levels. Tremblay et al. (2019) reported that group-level OS, in addition to being positively related to collective affective commitment, also acts as a mediator for group-level helping behaviors. In their repeated assessment on POS and extra-role performance at a three-year interval, Chen et al. (2009) found evidence that POS leads to extra-role performance. Of note is that among these studies, both Tremblay et al. (2019) and Li et al. (2017) aggregated individual-level POS into group/team-level POS for analysis. In view of the above findings, we posited that high POS and SOS should help the teachers and school attain its objectives by exhibiting high OCBs:

*H1a*: POS will be positively related to OCB.

*H1b*: SOS will be positively related to OCB.

### SOS and individual-level JE

On the SET, employees with high POS tend to be more involved in their jobs and organizations to help achieve organizational goals as part of the SET norm of reciprocity (Byrne and Hochwarter, 2008). POS conveys the organization’s evaluation of employees’ efforts and satisfies their needs for respect and recognition, which could also promote employees’ interest, thereby increasing their job engagement (Eisenberger and Stinglhamber, 2011). Therefore, the support from organizations, supervisors, or managers can often promote JE. Some studies have investigated the impact of POS on job engagement, for example, Jonsdottir and Kristinsson (2020) observed a significant positive relationship between supervisors’ active-empathetic listening, a manifestation of organizational support, and employees’ job engagement. Imran et al. (2020) found that POS significantly and positively affects employee s’ job engagement. Managerial support for development (Kumar et al., 2018) can increase intention to engage in work, and also in the study conducted by Gillet et al. (2013) reported the positive relationship between POS and job engagement characterized by vigor, dedication, and absorption. Saks (2006) found POS was positively related to job engagement and organizational engagement (i.e., participation in one’s role as a member of an organization). Structural equation modeling results on the relationship between POS and JE of bank employees revealed that POS fosters JE (Karatepe and Aga, 2016). Similarly, Kinnunen et al. (2008), Rahman et al. (2020), and Yang et al. (2020) found that POS was positively associated with key dimensions of job engagement. Similar findings of their positive relationship were also obtained by Caesens and Stinglhamber (2014) and Jin and McDonald (2017). In line with the above findings, we posited that high POS and SOS can help teachers demonstrate high JE:

*H2a*: POS will be positively related to JE.

*H2b*: SOS will be positively related to JE.

### SOS, individual-level JE, and individual-level OCB

Many previous studies have found the positive relationship between POS and OCB (Masterson et al., 2000; Suliman and Al Obaidli, 2013). However, some studies also pointed out that there are mediating variables between POS and OCB (Islam et al., 2014; Muhammad, 2014). Among them, JE could be considered as a mediating variable between POS and OCB. Alshaabani et al. (2021) supported that POS is positively related to OCB and JE is also a strongly mediating variable in the relationship. Using hierarchical linear modeling (HLM), Zhong et al. (2016) analyzed the relationships between individual-level POS, JE, and OCB and found that POS can have an indirect impact on OCB through the mediation of JE. Sulea et al. (2012) showed that POS and conscientiousness are positively related to OCB through employees’ JE. Other studies also supported the mediating role of JE (Karatepe, 2013; Shams et al., 2020).

This study analyzes the social interaction between the school and its teachers on the basis of individual-level POS. Further analysis is made with SOS for comparison. In this study, teachers’ JE would play a mediating role in the relationship between the effects of POS and SOS on OCB:

*H3a*: POS will be indirectly but positively related to OCB through the mediation of JE.

*H3b*: SOS will be indirectly but positively related to OCB through the mediation of JE.

## Materials and methods

### Sample

The study sample comprised elementary school teachers in Taiwan. There are differences in the size of schools and the number of teachers in various regions of Taiwan, in order to make the sampling more representative, 121 schools were selected from 11 counties distributed in northern, eastern, southern, and central Taiwan through stratified random sampling. Then according to the school size, different number of teachers were selected. Ten teachers were selected from the large schools, 8 from the medium-sized schools, and 6 from the small schools. A total of 1,040 (121 schools) questionnaires were sent out, of which 752 (98 schools) were returned, making up an overall response rate of 72.31%. After eliminating 22 partial or incomplete responses, 730 responses were valid for analysis, giving an effective response rate of 70.19%. Of the 730 respondents, 36.3% were male. The majority were aged 31–40 years (44.8%) and 41–50 years (41.1%) Their average teaching experience was 15.2 years.

### Measures

The self-report questionnaire administered for collecting empirical data comprised a total of 31 items (see Appendix 1), to which respondents were asked to indicate their agreement using a seven-point Likert scale (1 indicates strongly disagree; 7, strongly agree). These items for testing different variables were adopted or adapted from scales validated by empirical studies in organizational research. The 5 items under POS were adopted from those used by Eisenberger et al. (2001). The 9 items under JE were taken from the scale used by Rich et al. (2010) and are categorized into three dimensions, representing physical, cognitive, and emotional engagement. The 17 items under OCB were extracted from the scale developed by Somech and Ron (2007) and listed under five dimensions, namely altruism, conscientiousness, sportsmanship, courtesy, and civic virtue.

The above scales used were originally in English. For the questionnaire to be administered to respondents in Taiwan, this study applied the back-translation technique (Brislin, 1980) to come up with a version in Traditional Chinese. First, the forward translation from English to Traditional Chinese was performed by a bilingual Taiwanese translator. Six experts with related background in organizational research were invited to review and amend the translated items to ensure consistency in meaning and connotations with the original. Then, another bilingual Taiwanese translator back-translated the questionnaire into English. Comparison was made with the original English version to check further if any item or term discrepancy had occurred. Moreover, 10 scholars with school administration expertise and 6 elementary education practitioners were invited to review the descriptions in the items to confirm their correctness and relevance in the school organizational context.

Confirmatory factor analysis (CFA) was performed to examine the validity and reliability of the scales used in this study and the descriptive statistics of the variables were calculated. The required fit statistics are RMSEA and SRMR <0.08 and CFI and TLI > 0.90 (Hu and Bentler, 1999).

***POS***. For the 5-item construct POS, the results of first-order CFA are χ^2^ = 19.90 (*df* = 5, *p* < 0.001), RMSEA = 0.06, SRMR = 0.01, CFI = 0.99, and TLI = 0.99, factor loadings ranging between 0.80 and 0.89, and Cronbach’s α = 0.93, indicating good validity and reliability. To justify the aggregation of SOS, within-group agreement measure *r*_wg_ was calculated (Klein and Kozlowski, 2000). Results of *r*_wg_ obtained were all higher than 0.90, which exceeds the required.70 (Epitropaki, 2013), revealing high consistency within the group.

***JE***. For the 9-item construct JE, the results of second-order CFA are χ^2^ = 67.24 (*df* = 24, *p* < 0.001), RMSEA =0.05, SRMR = 0.02, CFI = 0.99, and TLI = 0.99, factor loadings ranging between 0.87 and 0.94, and Cronbach’s α of the three dimensions = 0.92, 0.93, and.94, also indicating good validity and reliability.

***OCB***. For the 17-item construct OCB, the results of second-order CFA are χ^2^ = 392.26 (*df* = 114, *p* < 0.001), RMSEA = 0.06, SRMR = 0.04, CFI = 0.97, and TLI = 0.96, factor loadings ranging between 0.70 and 0.94, and Cronbach’s α of the five dimensions = 0.93, 0.84, 0.81, 0.91, and 0.84, also indicating good validity and reliability.

### Analyses

The relationships between the variables were tested using multilevel structural equation modeling (MSEM) with Mplus Version 7. Not only is MSEM more flexible, it is superior to HLM in handling latent variables and to SEM in dealing with complex relationships on different levels. Moreover, Preacher et al. (2010) have demonstrated the advantage and applicability of MSEM in assessing multilevel relationship and mediation. Furthermore, the maximum likelihood-based estimation on Mplus yields estimates with standard errors that are robust to non-normality and non-independence of observations and can adjust the goodness-of-fit estimates (Muthén and Muthén, 2012). The mediating effect between latent variables was examined using delta parameterization of Mplus (MacKinnon, 2008; Muthén and Muthén, 2012).

## Results

### Descriptive statistics

Table 1 displays descriptive statistics and zero-order correlations. The correlation coefficients between POS and JE (*r* = 0.38, *p* < 0.001) as well as that between POS and OCB (*r* = 0.39, *p* < 0.001) indicate moderate positive relationship while that between JE and POS (*r* = 0.77, *p* < 0.001) reveal a strong positive relationship.

**Table 1:** Summary statistics and correlation of individual- and school-level variables.

Variables	M	SD	1	2	3
Individual-level					
1. Perceived organizational support (POS)	5.04	1.00			
2. Job engagement (JE)	5.80	0.76	0.38^***^		
3. Organizational citizenship behavior (OCB)	4.98	0.61	0.39^***^	0.77^***^	
School-level					
School organizational support	5.05	0.47			

*n* = 730 at individual-level; *n* = 98 at school –level.

*** *p* < 0.001.

### SEM analyses

**Figure 2:**
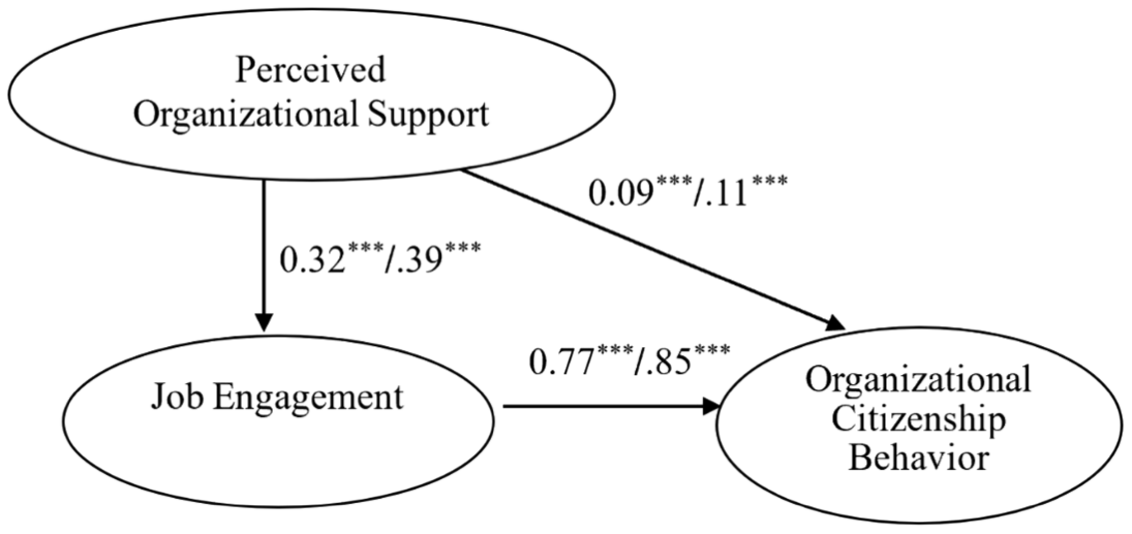
SEM analysis on individual-level POS, JE, and OCB. Note: unstandardized estimate/standardized estimate ***p < 0.001.

First, the goodness-of-fit statistics are CFI = 0.98, TLI = 0.97, RMSEA = 0.06, and SRMR = 0.04, indicating good model fit. Figure 2 shows the SEM results on the impact of teacher’s POS at individual level on JE and OCB. As can be seen, POS has direct and positive relationships with both JE (β = 0.39, *p* < 0.001; H2a supported) and OCB (β = 0.11, *p* < 0.001; H1a supported); similarly, JE is directly and positively related to OCB (β = 0.85, *p* < 0.001). As for the meditator role of JE, POS does have an indirect and positive impact on OCB through JE. In view of the indirect impact of POS on OCB *via* JE (β = 0.33, *p* < 0.001; H3a supported) exceeding the direct impact of POS on JE (β = 0.39, *p* < 0.001) and JE on OCB (β = 0.85, *p* < 0.001), JE serves only as the partial mediator.

### MSEM analyses

Analysis performed using MSEM also showed good model fit results: CFI = 0.98, TLI = 0.97, RMSEA = 0.03, and SRMR = 0.03/ 0.08 (within/between groups). Table 2 lists the estimates obtained using MSEM with SOS as the independent variable, individual-level JE as the mediator variable, and individual-level OCB as the criterion variable.

**Table 2:** Parameter estimates obtained using MSEM.

Variables	Unstandardized estimate	S.E.	Est./S.E.	standardized estimate
Within level				
Physical engagement → JE	1.00	-	-	0.90^***^
Emotional engagement → JE	1.07	0.05	21.77	0.81^***^
Cognitive engagement → JE	1.01	0.04	25.83	0.88^***^
Altruism → OCB	1.00	-	-	0.78^***^
Conscientiousness → OCB	1.11	0.06	19.19	0.81^***^
Sportsmanship → OCB	0.73	0.07	11.31	0.49^***^
Courtesy → OCB	0.92	0.06	15.16	0.67^***^
Civic virtue → OCB	1.08	0.07	16.62	0.72^***^
JE → OCB (*a*_W_)	0.82	0.05	17.70	0.89^***^
Between level				
S1 → SOS	1.00	-	-	0.86^***^
S2 → SOS	1.18	0.12	9.81	0.89^***^
S3 → SOS	1.38	0.14	9.94	0.92^***^
S4 → SOS	1.17	0.13	9.38	0.89^***^
S5 → SOS	1.27	0.11	11.24	0.90^***^
Physical engagement → JE	1.00	-	-	0.98^***^
Emotional engagement → JE	1.14	0.17	6.82	0.82^***^
Cognitive engagement → JE	1.19	0.10	11.75	0.99^***^
Altruism → OCB	1.00	-	-	0.91^***^
Conscientiousness → OCB	1.11	0.26	4.28	0.83^***^
Sportsmanship → OCB	1.29	0.41	3.15	0.94^***^
Courtesy → OCB	1.51	0.35	4.33	0.99^***^
Civic virtue → OCB	1.50	0.36	4.13	0.89^***^
SOS → JE (*b*_B_)	0.32	0.10	3.14	0.55^***^
JE → OCB (*a*_W_)	0.65	0.16	4.20	0.87^***^
SOS → OCB	0.06	0.05	1.17	0.13
Indirect effects				
*a*_W_ × *b*_B_	0.26^**^	0.08	3.12	0.48^**^

*N* = 730/98 (respondents/schools).

** *p* < 0.01;

*** *p* < 0.001.

**Figure 3:**
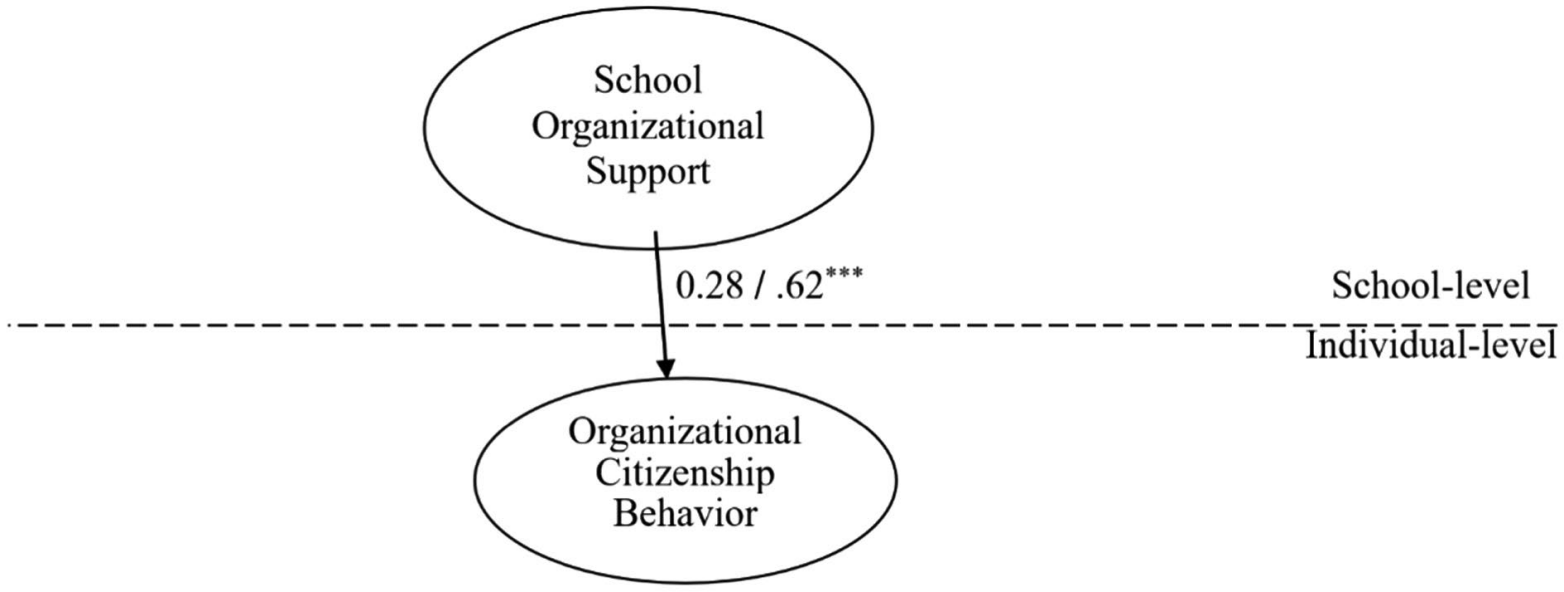
Cross-level SEM analysis on SOS and individual-level OCB. Note: unstandardized estimate/standardized estimate ***p < 0.001.

As suggested by Baron and Kenny (1986), to understand the mediating role of JE in the cross-level model, the direct impact of SOS on individual-level OCB must first be analyzed. Results shown in Figure 3 reveal a direct and positive relationship between these two variables (β = 0.62, p < 0.001).

**Figure 4:**
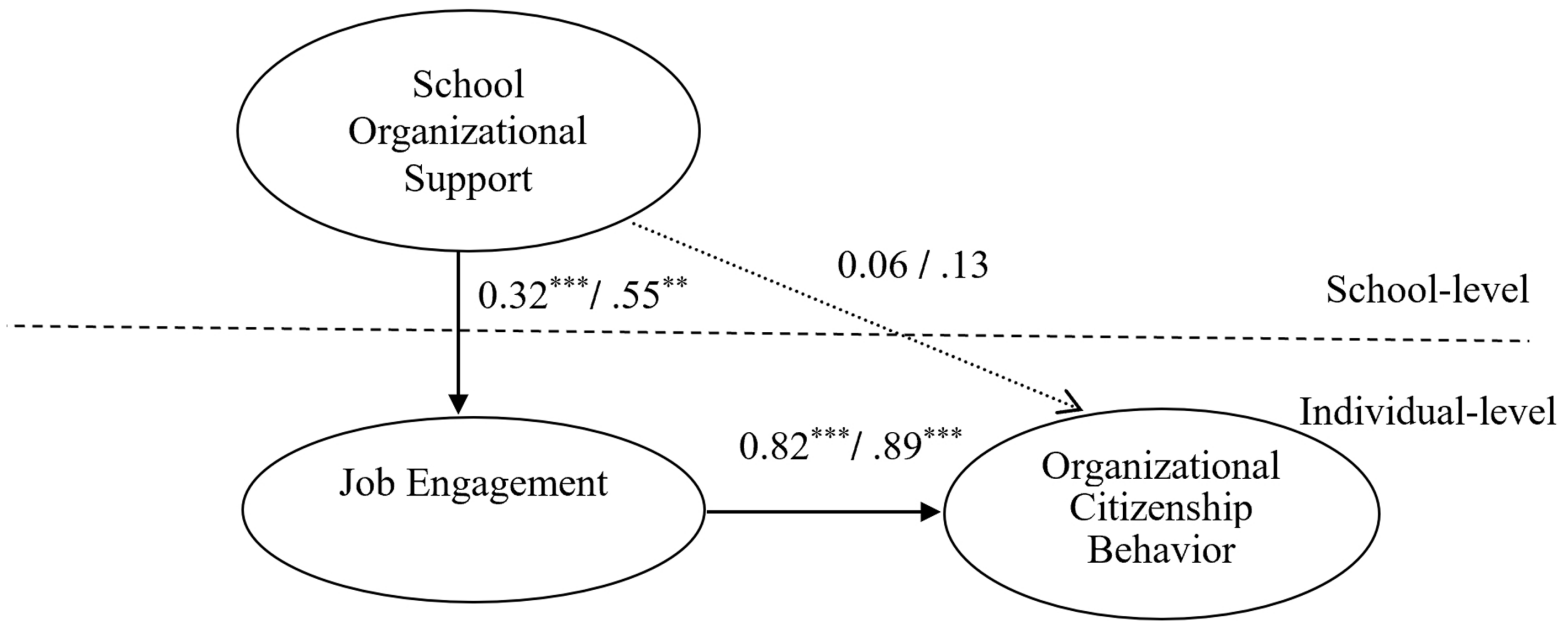
MEM analysis on SOS, individual-level JE and individual-level OCB. Note: unstandardized estimate/standardized estimate ***p < 0.001, **p < 0.01.

Cross-level analysis results obtained using MSEM are illustrated in Figure 4. As can be seen, SOS is directly and positively related to teachers’ JE (β = 0.55, *p* < 0.001; H2b supported), and JE has direct and positive relationship with their OCB (β = 0.89, *p* < 0.001). While SOS has no significant positive relationship with individual-level OCB (β = 0.13, *p* > 0.05; H1b not supported), it does have a positive impact on OCB through the mediation of JE (β = 0.48, *p* < 0.001; H3b supported). Comparison with Figure 3 reveals the change in effect of SOS on individual-level OCB from significant (β = 0.62, *p* < 0.001) to insignificant (β = 0.13, *p* > 0.05), thus implying full mediation effect of JE when SOS on individual-level OCB.

## Discussion and conclusion

### Partial mediation of JE between individual-level POS and OCB

The direct and positive relationship between POS and OCB at individual level revealed by SEM is consistent with previous findings by Chen et al. (2009), Ahmad and Zafar (2018), and Zhang et al. (2017). This result evidences the OST that teachers will exhibit OCB in return for POS manifested in the school’s appreciation of their contributions and concern for their well-being. Moreover, the positive relationship between teachers’ POS and their JE echoes the results obtained by Karatepe and Aga (2016), Caesens and Stinglhamber (2014), Jin and McDonald (2017), and further shows that the “social exchange” stimulated by POS, according to the OST, involves not only the behavioral but also the cognitive aspect, as in the case of JE examined in this study. Owing to the positive relationship between JE on OCB, JE motivated by POS serves the mediating role in promoting OCB.

### Full mediation of JE between SOS and individual-level OCB

A specific focus on this research is the impact of aggregated POS at the school level. While the insignificant relationship between SOS and teachers’ OCB has also been reported by Li et al. (2017), the positive impact of SOS on JE has not been observed in other empirical studies. Nevertheless, it is conceivable that greater SOS would create an encouraging atmosphere for greater appreciation of the teachers’ hard work and efforts, which would, in turn, enhance their JE. Moreover, SEM analysis at individual level showed a direct and positive relationship between JE and OCB. Hence, MSEM analysis on the relationship of SOS with individual-level JE and OCB revealed the full mediation effect.

### Individual-level POS versus SOS

This research examines the impact of OS at both individual and school levels on teachers’ JE and OCB. Conceptually, the two levels of OS are similar as they are both rooted in the OST, while their difference lies in one being closer to the organizational atmosphere and the other being personal cognitive perception. With regard to assessment, the same scale was used in this study for both levels, only with the individual perception of OS aggregated into a single value to represent the SOS. In terms of influence, individual-level POS has greater impact on OCB than SOS. Similar empirical evidence of such difference in impact has not been reported in the field of education but variation in results obtained using individual-level and group-level OS has been observed in the management study conducted by Li et al. (2017). In other words, different levels of group-level OS used in the analysis may influence the final results.

### JE as significant mediating variable

In-depth investigation on meaningful mediating variables is very important for quantitative research. In this study, JE was found not only to be positively related to both individual-level POS and SOS, it also plays a significant mediating role in the relationship between OS of both levels and OCB. In other words, teachers who are physically, cognitively, and emotionally engaged tend to behave more positively toward the school beyond their formal job requirement, thus exhibiting OCB.

## Implications for school practice

New tasks and roles shouldered by teachers as a result of incessant reforms in education have also aroused growing attention and emphasis on their OCB. Of particular interest are factors promoting teachers’ OCB that can help make up deficiency in the formal education system and educational resources. The present findings evidence that school organizational support is conducive to enhancing JE and encouraging OCB. This is of particular meaning in Taiwan where motivation and incentives for teachers to take up more duties or make extra efforts are lacking in the education system. As implied by the results, the school should show greater appreciation of the teachers’ contribution and concern for their well-being, thus creating a supportive organizational atmosphere that can motivate teachers to have greater job engagement. The emphasis in this study on the aggregated SOS highlights the importance of an organizational culture which will persist albeit personnel changes.

## Limitations and future research

This study has several limitations. First, responses of the self-report questionnaire were collected from the participating teachers at a single time point, thus common method variance may be a concern. Second, the scales adopted are those used in management research, indicating tool deficiency in educational organization research. Hence, there should be greater effort devoted to the development of assessment tools applicable to the culture and characteristics of the domestic educational context.

## Data availability statement

The original contributions presented in the study are included in the article/supplementary material, further inquiries can be directed to the corresponding author.

## Author contributions

C-CH is responsible for the overall research architecture design, as well as research conclusions. W-CC was responsible for data analysis and discussion of research results. H-CY and H-CL were responsible for data and literature collection. All authors contributed to the article and approved the submitted version.

## Funding

This work was supported by the Ministry of Science and Technology, Taiwan.

## Conflict of interest

The authors declare that the research was conducted in the absence of any commercial or financial relationships that could be construed as a potential conflict of interest.

## Publisher’s note

All claims expressed in this article are solely those of the authors and do not necessarily represent those of their affiliated organizations, or those of the publisher, the editors and the reviewers. Any product that may be evaluated in this article, or claim that may be made by its manufacturer, is not guaranteed or endorsed by the publisher.

## Appendix 1. Questionnaire

Perceived organizational support (POS)/school organizational support (SOS).

1. The school recognizes my extra efforts.
2. The school takes my complaints or opinions seriously.
3. The school really cares about my well-being.
4. The school notices my dedication to work.
5. The school cares about my job satisfaction.

Job engagement (JE)

1. I exert my full effort to my job.
2. I try my hardest to perform well on my job.
3. I strive as hard as I can to complete my job.
4. I am enthusiastic in my job.
5. I feel energetic at my job.
6. I feel positive about my job.
7. At work, my mind is focused on my job.
8. At work, I pay a lot of attention to my job.
9. At work, I am absorbed by my job.

Organizational citizenship behavior (OCB)

1. I help other teachers who have been absent.
2. I help others who have heavy workloads.
3. I willingly help others who have work-related problems.
4. I am always ready to lend a helping hand to others around me.
5. I do not take extra breaks.
6. I obey school rules and regulations even when no one is watching.
7. I am one of the most conscientious teachers in the school.
8. I believe in giving an honest day’s work for an honest day’s pay.
9. I tend to make “mountains out of molehills.”
10. I always find fault with what the school is doing.
11. I am the classic “squeaky wheel” that always needs greasing.
12. I am mindful of how my behavior affects other people’s jobs.
13. I do not abuse the rights of others.
14. I try to avoid creating problems for colleagues.
15. I attend meetings that are not mandatory, but are considered important.
16. I attend functions that are not required but help the school image.
17. I keep abreast of changes in the school.
